# Pharmacological targeting of gastric mucosal barrier with traditional Chinese medications for repairing gastric mucosal injury

**DOI:** 10.3389/fphar.2023.1091530

**Published:** 2023-06-08

**Authors:** Xueyan Jia, Yihuai He, Lin Li, Delin Xu

**Affiliations:** ^1^ Department of Cell Biology, Zunyi Medical University, Zunyi, China; ^2^ Department of Medical Instrumental Analysis, Zunyi Medical University, Zunyi, China; ^3^ Department of Infectious Diseases, The Affiliated Hospital of Zunyi Medical University, Zunyi, China

**Keywords:** traditional Chinese medications (TCMs), repair, gastric mucosal barrier, pharmacological mechanism, review

## Abstract

**Introduction:** The gastric mucosa (GM) is the first barrier and vital interface in the stomach that protects the host from hydrochloric acid in gastric juice and defends against exogenous insults to gastric tissues. The use of traditional Chinese medications (TCMs) for the treatment of gastric mucosal injury (GMI) has long-standing history and a good curative effect. Whereas there are poor overall reports on the intrinsic mechanisms of these TCM preparations that pharmacology uses to protect body from GMI, which is crucial to treating this disease. These existing reviews have deficiencies that limit the clinical application and development of both customary prescriptions and new drugs.

**Methods:** Further basic and translational studies must be done to elucidate the intrinsic mechanisms of influence of these TCM preparations. Moreover, well-designed and well-conducted experiences and clinical trials are necessary to ascertain the efficacy and mechanisms of these agents. Therefore, this paper presents a focused overview of currently published literature to assess how TCMs action that facilitates the cures for GMI. It offers a whole train of current state of pharmacological evidence, identifies the pharmacological mechanisms of TCMs on GM, and highlights that remarkable capacity of TCMs to restore GM after damage.

**Results:** These TCMs preparations promote the repair of multicomponent targets such as the gastric mucus, epithelial layer, blood flow (GMBF) and lamina propria barrier.

**Summary:** Overall, this study has summarized the essential regulatory mechanisms and pharmacological efficacy of TCMs on new and productive therapeutic targets.

**Discussion:** This review provides an avenue for studying various drugs with potentially promising effects on mucosal integrity, as well as subsequent pharmacological studies, clinical applications, and new drug development.

## 1 Introduction

Gastric mucosal injury (GMI) is the leading cause of most gastric-related diseases worldwide. It is characterized by a high morbidity and relapse rate, which affects 5%–10% of the world’s population at some point in life that occurs according to clinical and experimental data. Due to its diversity of etiologies and complexity of condition, along with its pathological characteristics usually involve all levels of the mucosa, making its treatment and complete cure very challenging, so much so that some researchers have referred to it as the new plague of the 21st century (Sánchez-Mendoza, et al., 2022; O'Malley, 2003). However, predisposing various gastric mucosal lesions, of whatever types or degree of assault, originally show the stages-specific, advances-rapid, and involvement-extensive response in an orderly stepwise progression (Hagen, 2022). The gastric mucosa (GM) employs two kinds of overlapping, depth-dependent and programmed mechanisms to effectively respond to diverse types of irritants and injuries affecting the stomach. Superficial injury heals through surface cells accompanied by histopathological alterations. Deeper, more often chronic, injury/inflammation, elicits glandular histopathologic alterations (Goldenring and Mills, 2022). And the development route was refined into four cascade stages based on the pathophysiological, histopathological, and clinical features of GMI induced by various risk factors. These stages also take into consideration the degree of GMI and the depth and scope of involvement. Within the detailed stages are as follows: the level of mucus infection, the phase of surface epithelial cell lesions, a decline in gastric mucosal blood flow (GMBF) and the stage of lamina propria lesion (Wang, et al., 2022).

At the outset, the loss of O-linked mucins (MUCs) from the mucus layer is the first step in the occurrence and advancement of GMI. Studies have verified that MUCs and wound-healing proteins (such as TFF2), as well as other transcriptional alterations are induced by attack factors (Goldenring and Mills, 2022). Specifically, changes and transformation occur in the physiological and morphology characteristics of mucous cells when interfered and blocked by *Helicobacter pylori* (*H. pylori*). This manifests as the disappearance of oval and spherical particles containing mucus that are wrapped by the bounded membrane in the cytoplasm around the nucleus of surface mucus cells, while the cytoplasm undergoes spiderweb-like vacuolar degeneration (Wang, et al., 2022). This is immediately followed by altered polarity, migration, invasive growth, and pro-inflammatory and proliferative responses of the epithelial cells, thus resulting in superficial mucosal lesions (Pimentel-Nunes, et al., 2022). These alterations are closely associated with apoptosis, necrosis, autophagy, and other modes of cell death in the epithelial tissue. Subsequently, most focal injuries tend to occur within the interior of the epithelium, where broken blood vessels are more prevalent due to the dysregulation of vascular endothelial cells (ECs), resulting in reduced GMBF, ischaemia, hypoxia and tissue necrosis. Relevant studies have indicated that tissue damage caused by ischemia lasting for more than 60 min induces the loss of barrier function in the GM (Eduardo, et al., 2021). And the damaging agents that accompany with GMBF can reach the lamina propria, causing gastric hydrochloric acid-related disorders mediated by parietal cells that penetrate into the interior of the lamina propria. As a result, GMI is deeply exacerbated by counter diffusing hydrochloric acid and diffuse injury occurs (Zhao, et al., 2022a).

Beyond that, GMI is an ever-expanding disease which not only involves potentially serious complications such as bleeding or perforation, with a high risk of mortality (Sverdén, et al., 2019), but also provokes the likelihood of developing digestive system diseases, including gastroesophageal reflux disease, intestinal ulcers, and other peptic-related disorder (Isik, et al., 2022). The mechanisms responsible for this particularly pathogenic and idiopathic condition of GMI are mainly attributed to the layer-by-layer involvement of autologous mucosal components. The classic treatment means for GMI also correspond to various barriers contained by GM. For example, the protective agent of magnesium aluminate is reflected in the mucous layer, while proton pump inhibitors (PPIs) and H2 receptor inhibitors are in alignment with the lamina propria. However, these chemicals inherently pose problems that may not be as effective for treating GM diseases, as they often have only one mode of action, unstable efficacy, strong drug resistance, significant side effects, and the need for repeated treatment cycles. Additionally, they can cause high levels of negative side effects, and result in strong drug resistance. Furthermore, after drug discontinuation, patients have a high chance of relapse, as much as 70%. There may also be other unforeseeable adverse effects that could arise as a result of patients taking these medications for a prolonged duration (Hu, et al., 2017). Thus, majority of western drugs are not suitable for long-term therapy in treating GMI. For example, histamine receptor blockers like cimetidine not only target histamine receptors but also androgen receptors (Manzato, et al., 2021). And ranitidine also has a series of side effects, including contamination with N-nitrosodimethylamine (NDMA) (Aldawsari, et al., 2021), shock (Russom, et al., 2021) and cancer (Cardwell, et al., 2021). In particular, ultra-dose use of PPIs, such as omeprazole and its derivatives, prolonged treatment duration between 1 and 5 years, and overprescription increase the risk of GI bleeding, pre-eclampsia, gestational diabetes, and preterm birth (Bolek, et al., 2019; Breddels, et al., 2022). Several studies have highlighted the pathogenic drawbacks of PPIs in the medical treatment of GMI, since PPIs irreversibly inactivate H^+^/K^+^-ATPase activity by binding to cysteine 813, thus immobilizing the enzyme in the E2 configuration (Shin, et al., 2011; Bruno, et al., 2019).

Therefore, exploration and elucidation for effective and productive drugs on efficient therapeutic targets of the gastric mucosal barrier using fundamental molecular means has become the overarching objective in the treatment of GMI. TCMs and their formulas, which include multiple products such as Chinese herbal medicines, fungi and insects share an excellent ability to repair mucosal damage. This ability is achieved through their own multi-molecules, multi-pathways, multi-targets and low toxicity that have been applied in the prevention and treatment of GMI for literally thousands of years. The treatment of stomach diseases using TCMs has a rich history dating back as early as two thousand years ago. This practice is documented in the Huangdi Neijing, which is considered the first Chinese medicine classic and contains thirteen prescriptions. Moreover, the four classics of TCMs all emphasize the significance of the stomach and its functions. Through numerous pioneering explorations and experiments, classical Chinese medicines and prescriptions for treating GMI have been passed down to the present day. These include Shaoyao-Gancao decoction (SGD), *Bletilla striata* (Thunb.) Reichb. f, *Dendrobium*, and many more. And TCMs have been clinically validated in the treatment of erosion, ulcers, and even gastric perforation mediated by damage to the gastric protective barrier. Even now, classical prescriptions and decoctions are still utilized for treating gastric mucosal diseases. For instance, the popular Weikangling medication is composed of peony and licorice decoction (Meng and Li, 2020). Therefore, TCMs can provide an important option for repairing the gastric mucosal barrier. However, the mechanisms by which they protect the body from GMI are not well understood that hinder the application of classic prescriptions and the development of TCMs, so insights into how TCMs repair the gastric mucosal barrier through biological network of host systems are therefore necessary. The research data documented that complex cross-linking among TCMs components ensure an all-encompassing defense strategy by reconstituting of GM and multi-targets. Then, this leads to the restoration of the multiple inner components of the gastric mucus, the epithelial layer, blood flow (GMBF) and the lamina propria barrier that act as protective agents against the acidic nature of the gastric juice.

## 2 Reconstitution of the gastric mucus barrier

As the first line of defense in protecting the GM, water and glycoproteins have been made in defining critical components of covering the surface of the mucus layer. So that it is influenced first by injuries along with exhibits high susceptibility, with resultant a reduction in biosynthesis and/or shedding of mucus. The restorative effect of various TCM preparations on the mucus layer can be targeted towards *O*-glycan cross-linked MUCs (including MUC1, MUC5AC, MUC6, etc.) and their components such as galactose (Gal), GalNAc, *N*-acetylglucosamine (GlcNAc, NAG), sialic acid (Neu5Ac), and fucose (Fuc) (Jin, et al., 2017).

### 2.1 Modulatory effects of TCMs on *O*-glycans cross-linked by gals

**FIGURE 1:**
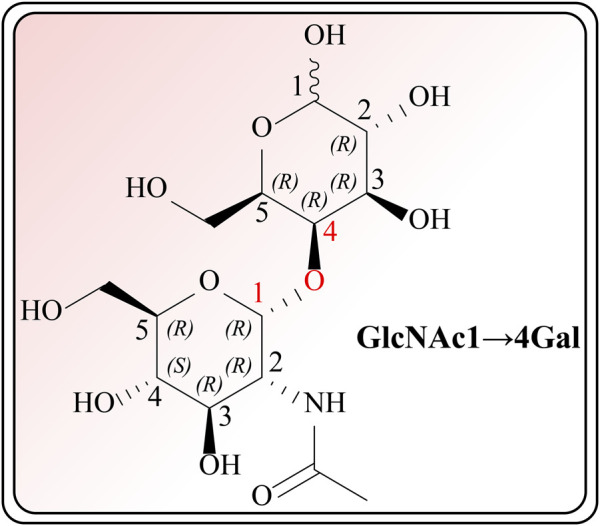
Chemical structure of GlcNAc α1→4Gal.

The therapeutic functions of swiftlet nest (Babji and Daud, 2021), snail secretion filtrates (Fan, et al., 2021) and other TCMs components with preventive and counteractive effects on spontaneous gastritis and gastric cancer are closely linked with gastric mucin *O*-glycosylated MUCs (Liu, et al., 2020; Pruss, et al., 2021). What calls for special attention is that in the absence of MUCs, spontaneous gastritis occurs (Suzuki, et al., 2022). During the investigation of O-glycan formation and its mechanism, studies on MUCs confirmed that validated their ability to agglutinate and intertwine with Gals in a β-galectin-dependent manner. This binding creates a strong and permeable mucus barrier, which enhances the immune resistance to inflammation and bacteria (Tamura, et al., 2020). Furthermore, it is acknowledged that Gals accordingly contain carbohydrate recognition domains with specific affinity for *N*-acetyllactosamine (LacNAc; Galβ1, 4GlcNAc, Figure 1), which have a critical impact on cell recognition, adhesion and signaling. These domains are markedly reduced in GMI (Vasta, 2012; Järvå, et al., 2020). Similar agglutinating effects are observed in plant-derived D-galactose lectins, such as frutalin (a lectin belongs to the JRL family derived from *Artocarpus incisa* L.). It provides gastric protection at a significantly low dose of 0.5 mg/kg and exhibits greater efficacy, far superior to cimetidine control group (100 mg/kg), therefore exhibiting favorable repair for mucosal wounds by their own cohesive properties (Abdon, et al., 2012).

While some Gals in MUCs (e.g., Gal-1, Gal-2) can defend against GMI, others (e.g., Gal-3, Gal-7, Gal-9) adhere to and agglutinate potentially pathogenic microorganisms and *O*-glycans on the surface of parasites. These Gals act additively or synergistically to elicit and infect surrounding tissues, producing inflammatory responses and even cancers (Wan, et al., 2021). Therefore, researchers believe that the existence of Gals may be caused by tissue damage and leakage, thereby identifying them as potential biomarkers of GMI. It has reported that *Cyperus conglomeratus* Rottb. Inhibits the expression of Gal-3 in a dose-dependent manner (25 mg/kg, 50 mg/kg, 100 mg/kg). Additionally, its regulation of the expression level of Gals is inversely proportional to the regulation of glycoprotein expression in the layer of gastric mucus cells. Furthermore, the study also confirmed that it can inhibit the expression of Gals up to 97.1%, which is higher than the 81.4% inhibition achieved by ranitidine (Elshamy, et al., 2020). Even so, subsequent surveys have suggested that short-chain *O*-glycans formed by Gals are one of the most potentially risky factors of GMI. They may even serve as a specific marker for evaluating the degree of GMI (Freitas, et al., 2019).

Therefore, the identification of the “self” *O*-glycan fraction and prevention of dramatic changes in carbohydrate specificity by Gals has become an urgent problem for TCMs to enhance the resistance to adhesion of pathogenic bacterial glycans. Studies have proven that quinic acid glycerates (which are isolated from *Tussilago farfara*) can eliminate MUCs that bind pathogens by inhibiting the activity of *N*-acetylgalactosaminyl transferases (GalNAc-Ts), which are the initiating enzymes for mucin-type *O*-glycosylation. Importantly, this inhibitory activity is specific to GalNAc-Ts without affecting the activity of *O*-GlcNAc-transferase (OGT) in normal mucosal tissues (Feng, et al., 2021).

### 2.2 Modulatory effects of TCMs on αGlcNAc via TFFs

MUCs formed by crosslinking of Gals are the primary constituent of mucin, with α1,4-linked GlcNAc (αGlcNAc) being the major load-bearing contributors to MUCs (Rudenko, et al., 2019). Consequently, sea buckthorn (*Hippophae rhamnoides* L.) displayed an excellent efficacy to thicken gastric mucus by enhancing β-*N*-acetylhexosaminidases (β-Hex) activity and is superior to the ranitidine group. It also accelerates the synthesis of *O*-glycans containing αGlcNAc in MUCs to improve the hydrophobicity of the mucus layer and preventing the diffusion of H^+^, so as to promote the neutralization of H^+^ and HCO_3_^−^ and reduce the H^+^ damage to GM (Zhang, et al., 2019). Mucus-thickening properties of TCMs are dominated via GlcNAc-α-1,4-Gal disaccharide recognized, reversibly and noncovalently cross-linked by its cognate ligand for TFFs that contain conserved disulfide-rich peptides (Järvå, et al., 2020). After the data analysis, it was found that the protective effects of the alcohol extract of *Amomum longiligulare* T.L. Wu are linked to the secretion of ɑGlcNAc mediated by TFF1. This secretion is responsible for enhancing the density, viscosity, and elasticity of mucus, thereby enabling it to resist the detrimental effects of oxidative stress. The sulfhydryl group present in TFF1 plays a crucial role in this process (Deng, et al., 2019; Heuer, et al., 2020).

Another study has reported that the total triterpenoids found in *Chaenomeles speciosa* can attenuate the cellular motility and invasiveness. In addition, they can increase the viscosity and elasticity of the inner insoluble gastric mucus barrier layer to benefit mucus cohesiveness through TFF2. These compounds act as a lectin with highly specific binding to ɑGlcNAc, particularly binding to a conserved *O*-linked carbohydrate moiety of MUC6 on its own (He, et al., 2019; Heuer, et al., 2019). However, the core oligosaccharide of many *H. pylori* strains bears the Glc-α-1,4-Gal branch, while the N-acetyl group of GlcNAc-α-1,4-Gal could facilitate accommodation in the TFFs binding site and make no interactions with the peptide (Järvå, et al., 2020). As bacteria are able to adhere to and penetrate the gastric mucus barrier by virtue of their similarity in the structure to gastric mucus, complete eradication of GMI implemented by TCMs and western medicines is still not possible. Therefore, further research is currently needed.

### 2.3 Modulatory effects of TCMs on sialic acid

Sialic acid is a component of glycoproteins that forms the terminal sugar chains found on the cell surface glycoproteins, which plays an important role in cell recognition, localization, and adhesion. Research has shown that RW0117 extracted from *Artemisia argyi* possesses a positive restorative effect on the thermal stability and hydrophobicity of the gastric mucus. This is, achieved by significantly upgrading the content of sialylated MUCs in *O*-glycan and slowing down the GMI-driven abscission of neutral mucins from the glands to the epidermal mucus layer due to GMI. As a result, it offsets the drains of mucus and provides shelter for epithelial cells (Shin, et al., 2021; Habashy, et al., 2022; Sharba, et al., 2022). The mechanisms of TCMs that mentioned to act on gastric mucus show a significant curative effect, thus highlighting the potential of TCMs in the treatment of mucus-related conditions.

## 3 Reconstitution of the epithelial barrier

A single layer of columnar epithelial cells, characterized by continuous and rapid renewal, covers the entire mucosal surface along with the periphery of the gastric fovea. The tight junctions (TJs), also known as the paracellular pathway between adjacent epithelial cells form a vital component of this epithelium (Lin, 2004).

### 3.1 TCMs that restore the resistance of the reconstituted gastric surface epithelial cells by signaling pathways

Homeostasis of the epithelial sheet is maintained through the balanced process of cell proliferation, differentiation, migration, and death (Zagami, et al., 2022). The disruption of the mucus layer creates an environment where pathological factors such as inflammation, oxidative stress of lipid membranes, pathogenic bacteria, and DNA damage, are intensified. This exacerbation results in the destruction of rapidly renewing epithelial cells, ultimately demolishing the innate immune barrier provided by those cells. Therefore, the key to the resistance of epithelial cells to death lies in a series of intracellular signaling pathways that are modulated by TCMs, including the MAPK, NF-κB, G protein coupled receptor (GPCR) and PI3K/Akt (Puebla, et al., 2016). *In vitro* experiments suggested that multiple TCMs components can regulate epithelial cell death modalities (Table 1), such as apoptosis (including endogenous mitochondrial pathway (Zhang, et al., 2020a; Xie, et al., 2020; Bae, et al., 2021; Yao, et al., 2021; Zhang, et al., 2021), FASR (Liang, et al., 2018), TNFR pathway (Hsieh, et al., 2022) and endoplasmic reticulum stress-induced apoptosis pathway (Ma, et al., 2022), necroptosis (Shu, et al., 2022), apoptosis (Lee, et al., 2020; Tian, et al., 2020), and pyroptosis (Zhang, et al., 2022) through these signaling pathways.

**TABLE 1:** Pharmacological effects of TCMs on the apoptosis and proliferation pathways of epithelial cells.

Cell death		Active compounds of TCMs	Source of TCMs	Experimental model	Repair mechanism	Reference
Apoptosis	Intrinsic mitochondrial pathway	β-sitostero 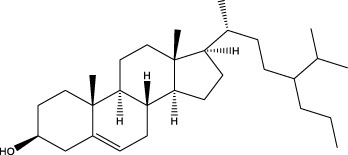	*Lotus plumule*	Ethanol causes mitochondrial Ca^2+^ overload, loss of membrane potential and ultimately induces GES-1 apoptosis	Ca^2+^↓, p-ERK↓, p-JNK↓, p-p38↓, restoring the antioxidant status of GES-1 cells and inhibiting apoptosis	Zhang, et al. (2021)
		Triterpenes 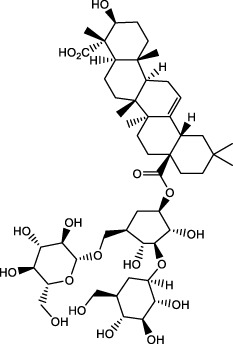	*Chaenomeles speciose* (Sweet) Nakai	IND triggers mitochondrial oxidative phosphorylation decoupling, membrane potential decomposition, permeability transition pore enlargement and cytochrome C release, which leads to apoptosis of RGM-1 cells	Apaf-1 mRNA↓, cytoplasmic C↓, thereby inhibiting mitochondria-mediated apoptosis	Zhang, et al. (2020a)
		Quercetin 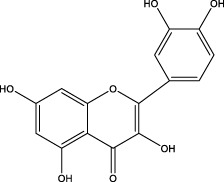	Onions, oranges, red wine, etc.	H_2_O_2_ causes increased mitochondrial permeability, MPTP opening, and descended MMP, ultimately causing apoptosis of GES-1 cells	Increased mitochondrial MMP in GES-1 cells via PI3K/Akt pathway	Yao, et al. (2021)
		β-carotene 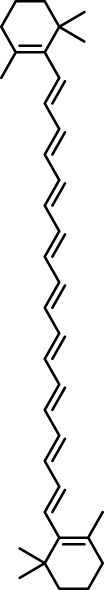	Carrots, Chinese wolfberry, kale, etc.	*H. pylori* decreases mitochondrial PPT, ATP in epithelial cells	PPAR-γ enhances nuclear translocation, upregulation of CAT, a PPAR-γ target gene, and increased mitochondrial viability	Bae, et al. (2021)
		Patchouli alcohol 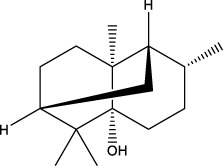	Patchou li	Ethanol entrains lipid peroxidation, inflammatory response and triggers GES-1 apoptosis pathway	p-ERK↓, p-JNK↓, p-p38↓, regulates the level of apoptosis factors downstream of GES-1	Xie, et al. (2020)
External death receptor pathway	FAS pathway	Patchoulene epoxide 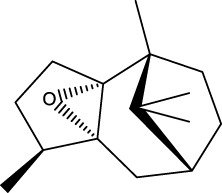	Patchouli alcohol	Apoptosis is triggered by GES-1 lipid peroxidation and inflammation infected by ethanol	NF-κB activation ↓, caspase-3 ↓, Fas ↓ and Fasl ↓	Liang, et al. (2018)
	TNF pathway	Quercetin 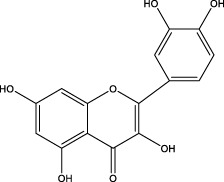	Onions, oranges, red wine	TNF-α irritates GES-1-cell injury	Regulation of proinflammatory TNFR-c-Src-ERK1/2-c-Fos and NF-κB pathways, MMP-9↓	Hsieh, et al. (2022)
	Endoplasmic reticulum stress-induced apoptosis pathway	Incense resin heartwood rich in 2-(2-phenylethyl) chromone	Chenxiang wood	Bile acid reflux stimulates endoplasmic reticulum stress, which eventually leads to GES-1 loss and disorder	Inhibition of apoptosis of GES-1 cells by regulating PERK/eIF2α/CHOP pathway	Ma, et al. (2022)
Necroptosis		Anthocyanin 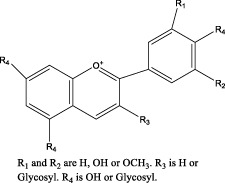	Blueberry	*H. pylori*-LPS activates MAPK/NF-κB signaling, which incentivizes necrosis of HGECs	Only the proliferation of injured HGECs was inhibited	Shu, et al. (2022)
		Cyanidin-3-O-glucoside 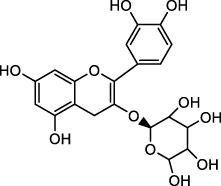	Anthocyanin	Abnormal synthesis of DNA predisposed by *H. pylori*-LPS	Modulation of TLR2 and TLR4 mediated NF-κB signaling, which guide the apoptosis of damaged HGEC	Tian, et al. (2020)
Autophagy		Astaxanthin 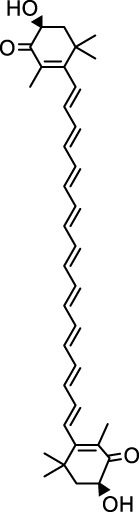	*Haematococcus pluvialis*, shrimp shell, etc.	*H. pylori* spurs autophagy and apoptosis in gastric epithelial cells	mTOR↓, activation of AMPK pathway	Lee, et al. (2020)
Pyroptosis		Astragaloside IV 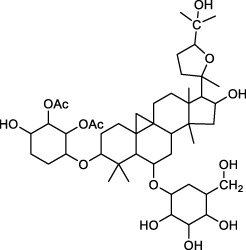	Radix Astragali	Enterovirus 71 (EV71) infection elicits GES-1-cell damage	By suppressing EV71 replication and release	Zhang, et al. (2022)

↑: upwards adjustment; ↓: downwards adjustment.

### 3.2 TCMs that restore structural collapse and dysfunction of the GMI

Members of the claudins (CLDNs) protein family, tight junction-associated marvel proteins (TAMPs) such as occludins (OCLNs) and junctional adhesion molecules (JAMs, which are member of the immunoglobulin superfamily), as well as cytosolic proteins exert their functional roles in forming barriers of gastric epithelium as integral proteins of TJs (Zeisel, et al., 2019). With higher levels of GMI, there is a lower expression of TJs, resulting in the loss of epithelial cells, increased permeability, and an imbalance of osmotic pressure. A myriad of clinical trials and experiments have demonstrated the positive effects of TCMs in repairing TJs, thereby attenuating the ecological dysregulation within the epithelial layer. TCMs achieve this by locking the gastric epithelial cells, transporting paracellular solutes, and acting as signaling hubs for gene expression. On one hand, TCMs promote the gene expression of TJs, which block epithelial cells and limits the movement of proteins within the plasma membrane. This help to repair mucosal damage and strengthen the barrier of TJs. For instance, the mRNA expression of TJ-associated genes, such as zona occludens proteins (ZO)-1 and ZO-2, is facilitated by grape skin extract. This helps in closing the gaps between gastric epithelial cells and subsequently contributes to the impairment of the epidermal barrier (Zhao, et al., 2022b; Thein, et al., 2022). Moreover, ZOs have the ability to interact with other TJs, including transmembrane proteins (OCLN, CLDNs, JAM-A) and F-actin, to create an interactional network of TJs. This network forms connecting plaques and links transmembrane components to the cytoskeleton. Cell model of peptic ulcer disease revealed that garlic oil in TCMs can scaled up the tension of the F-actin that drives ZO-1 protein towards the margin of the ulcer to speed up the process of gap-filling (Schwayer, et al., 2019; Kuna, et al., 2022). Meanwhile, certain western drugs may have an adverse effect on mucosal repair of GMI. A study has discovered that using lansoprazole can lead to a reduction of F-actin (Kuna, et al., 2022).

On the other hand, TCMs are able to regulate ion exchange, water flux, and paracellular solutes within the epithelial layer through CLDNs, AQPs, and others. This assists in maintaining cell polarity, normal morphology, physiological function, and osmotic pressure, ultimately leading to the steady-state balance of the epithelial layer. For example, CLDNs, particularly a deficiency in CLDN-18 within TJs, are believed to alter the anion flux that possibly cause an imbalance of intra- and extracellular ions (Günzel, 2017; Caron, et al., 2021). In contrast, the “ion-trapping” response results in the accumulation of damaging factors in the epithelium, especially when the GM is under attack (Kavvada, et al., 2006). Such conditions can be ameliorated by restricting the buildup of injury factors through the high permeability of gastric surface epithelial cells to the anionic form (Kavvada, et al., 2006). As disclosed above, RW0117 considerably enhanced the adhesion of sialylated MUCs to gastric epithelial cells, thereby increasing the high permeability of the anion in epithelial cells, while reducing the mucosal damage caused by the accumulation of harmful factors (Shin, et al., 2021; Sharba, et al., 2022).

Additionally, TJs can also be found beyond the confines of the TJs structure, where they function as cell signaling, trafficking and regulation of gene expression, which serve as a hallmark of TJs during the progression of epithelial-to-mesenchymal transition (Zeisel, et al., 2019). The aforementioned epithelial layer dysfunction and acquisition studies provide evidence that TCMs regulate the proliferation and apoptosis of gastric epithelial cells, thereby enhancing the therapeutic ability of the epithelial sheet. TCMs also modify the expression of ligand protein to strengthen the precautions and protection of the epithelial barrier.

## 4 Repairs to the GMBF barrier

**FIGURE 2:**
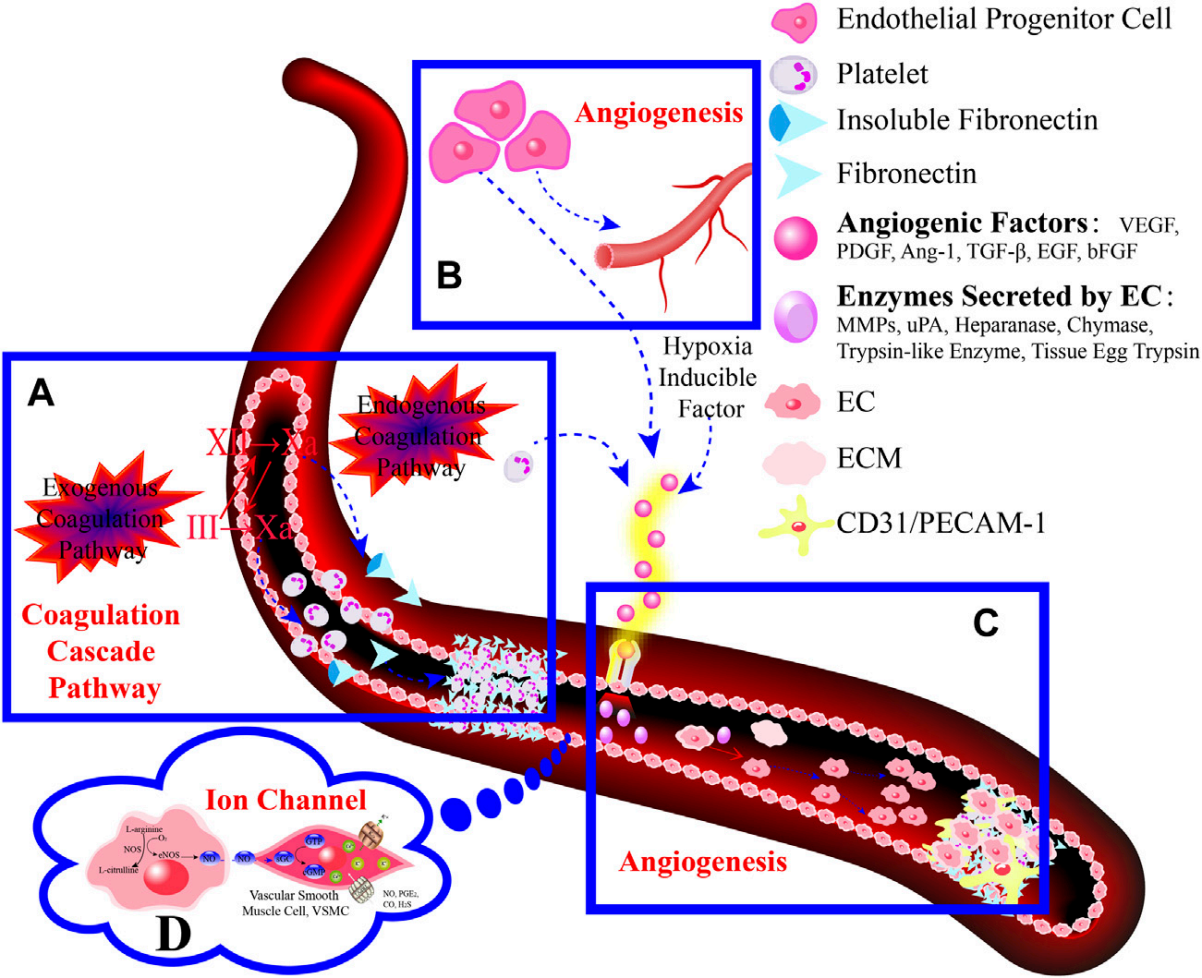
TCMs can repair vascular injury by promoting haemostasis **(A)**, vasculogenesis **(B)** and angiogenesis **(C)**, as well as improving I/R **(D)**. **(A)**. Coagulation cascade pathway. **(B, C)**. The process of angiogenesis. Angiogenesis is initiated by the release of proangiogenic factors, including VEGF and PDGF, from vessels that have been suggested to injury and hypoxia. These factors bind to receptors on ECs, causing the activating the ECs. Subsequently, the ECs secrete various proteases that activate TGF-β and b-FGF, promoting vascular growth and migration. Meanwhile, proteases degrade the basement membrane, allowing ECs to migrate and proliferate in the presence of adhesion factors like CD31/PECAM-1 (platelet-endothelial cell adhesion molecule). Eventually, neovascularization is achieved. **(D)**. Endogenous substances released from ECs alleviate inflammation.

The GMBF forms an essential mechanism for exchanging oxygen, carbon dioxide, nutrients, gastrointestinal (GI) hormones and other solutes among the components of the GM at the sub-epithelial level. This mechanism is wrapped by blood vessels that consist of ECs located deep within the epithelial layer. It ensures the renewal and maintenance of the structure and physiology of each tissue, and it also has metabolic activity. This ability that could be of production in various endogenous basic mediators in mucosa, such as PGE_2_, leukotrienes, procoagulants, NO, ET, EGF and others. These mediators regulate the circulation in the stomach, which make it closely related to the pathogenesis and healing of GI lesions (Yi, et al., 2021). Numerous studies have demonstrated that TCMs have therapeutic effects on the GMBF barrier, which can alleviate blood circulation imbalances and vascular dysfunction. This involves various processes (Figure 2) such as angiogenesis, activation of coagulation, and inhibition of the inflammatory progression during angiogenesis.

### 4.1 Mechanism and efficacy of TCMs in the treatment of GMBF

The migration of ECs is the most direct and important way used to accelerate the process of angiogenesis by repairing vascular injury (Tarnawski, et al., 2014). Among them, the precursor signal pathway, specific cytokines, biomarkers of ECs, and key enzymes that promote the growth and migration secreted by ECs are all crucial therapeutic targets of TCMs for regulating the repair of the GMBF. Clinical trials indicated that CD34, typical marker for endothelial progenitor cells, can be inspired by Kangfuxin (KFX) through the p38/NF-κB pathway, which contributes to angiogenesis and stimulates the formation of ECs in ischemic and hypoxic vessels. The degree of ulcer wounds healing was higher in the KFX group than in the conventional drug group that received rabeprazole sodium 1 month after surgery. Additionally, ulcer wounds in the KFX groups were completely healed 2 months after operation (Ahluwalia, et al., 2017; Tian, et al., 2021). Furthermore, studies have shown that *Artemisia argyi* is capable of upregulating the levels of VEGF and PDGF to expedite the formation of ECs. This is achieved by cooperatively regulating the HIF-1 signaling pathway (Wang, et al., 2019). Meanwhile, studies have shown that TCMs contribute to the activation of ECs that can secrete a variety of vital proteases such as matrix metalloproteinases (MMPs), uPA, tPA, PAl-1, COXs, and eNOS. This activation promotes the proliferation and migration of the ECs, which then speeds up revascularization (Tarnawski and Ahluwalia, 2021). A correlation study proved that EC proliferation and migration are elicited in response to MMP-2, which degrades the extracellular matrix (ECM) through *p*-cymene and rosmarinic acid. And both *p*-cymene and rosmarinic acid significantly reduced the lesion area of the GM in a dose-dependent manner, with the same dose and better efficacy than that of carbenoxolone group. In addition, the 14-day oral toxicity survey revealed that there were no changes in the weight of heart, liver, spleen, and kidney, as well as the parameters of biochemical and hematological evaluation (Formiga, et al., 2021).

Last but, more importantly, the angiogenic marker CD31/platelet endothelial cell adhesion molecule (PECAM)-1, which suggests existence of the ECs layer, can be elevated by *Panax notoginseng* to enhance the production of ECs thrombin in the fibronectin network (Sonnemann and Bement, 2011; Li, et al., 2021). In addition to stimulating the formation of ECs, TCMs can also activate the coagulation cascade pathway at the onset of angiogenesis to aid in the recruitment and management of ECs necessary for angiogenesis. Studies have suggested that the application of *Macaranga barteri* Müll.Arg (Euphorbiaceae) dose dependently prevented gastric lesion formation, which may be associated with facilitation on the proliferation and migration of platelets that release pro-vascular growth factors (Wallace, et al., 2006; Abayli and Gencdal, 2019; Ehilé, et al., 2021). In particular, folic acid (which is derived from Chinese herbal medicine, secrets, and fungi, such as *Angelica sinensis* (Oliv.) Diels), *Oecophylla smaragdina*, and others) significantly enhance fibronectins and platelets that are sensitized by factor VIII (FVIII) to act as a medium for ECs to expedite the restoration of the microvascular network and promote ulcer healing (Ajeigbe, et al., 2022; Yamane, et al., 2022).

### 4.2 Mechanism and efficacy of TCMs in the treatment of GMBF during the inflammation phase

During the GMI and angiogenesis phase, reduced GMBF velocity, evoked by the broken blood vessels is recognized as an initiator and participant in the inflammatory proceedings (Sonnemann and Bement, 2011; Pilard, et al., 2022). One such alarming family of molecules is the damage-associated molecular patterns (DAMPs), which are endogenous, constitutively expressed proteins and peptides that are chemotactic and immune activating. These molecules are induced and expedited by reduced GMBF velocity and act as intercellular signals in defense. They interact with chemotactic and pattern recognition receptors (PRRs) to galvanize immune cells and enhance host defense (Yang, et al., 2017). Sustained activation of ECs due to immune reactions, which includes circulating pathogen-associated molecular pattern (PAMPs), DAMPs, cytokines, chemokines, complement proteins and ROS cause alterations in the vasoactive endogenous mediators such as intravascular NO, PGE_2_, CO, H_2_S, NP-SH, Hsp-70 and endothelial function (Sidahmed, et al., 2013; Pilard, et al., 2022). Therefore, achieving the restoration of the normal physiological function of ECs and regulating of GMBF is considered the foremost issue in returning to the normal GMBF velocity implicated in the physiology of the GI tract (Magierowski, et al., 2018).

Relevant research has showed that endogenous mediators can serve as therapeutic targets for the treatment of TCMs in endothelial dysfunction and reduced GMBF velocity, achieved through the management of TCMs on the expression of upstream and downstream components of these mediators. And some studies revealed that chemical or pharmacological donors (including HMOX-1, HMOX-2, CORM, Nrf2 and other inducible enzymes) along with downstream components (K^+^ channel, capsaicin receptor/TRPV1) (Omayoneac, et al., 2020) of these endogenous mediums are primary targets of TCMs. These TCMs aim to address changes in the disorganized expression of endogenous mediators, which can further exacerbate vascular and mucosal injury. This exacerbation can lead to endothelial dysfunction, inflammation, vascular ischemia-reperfusion (I/R), microcirculatory exhaustion and other injury responses (Reinke and Sorg, 2012). Studies have determined that there is crosstalk between TCMs and endogenous substances in the mechanism of GMI recovery, restoration of the GMBF, modulation of smooth muscle movement and accompanying inflammation (Magierowski, et al., 2018; Magierowska, et al., 2019; Korbut, et al., 2020). Examples include curcumin, demonstrate its ability to open K^+^ channels through NO, that is, magnified by the NO/sGC/GMP/K^+^-ATPase pathways. Additionally, its gastric protective effect was found to be similar in magnitude to that produced by omeprazole (Díaz-Triste, et al., 2014). Both *in vivo* and *in vitro* experiments, including acidified ethanol (HCl/EtOH), piroxicam (NSAID), water restraint stress (WRS) and chronic acetic acid induced gastric ulcer (GU), have shown that *Chrysophyllum cainito* can activate the EPs receptor in PGE_2_ to relieve blood vessels. This is facilitated by GPCR-cAMP and K^+^ channel hyperpolarization, resulting in a significant reduction of the area of the gastric lesion. The efficacy of this treatment is equivalent to that of classic medicines, such as carbenoxolone and cimetidine, which are used for treating GMI (Arunachalam, et al., 2019). Furthermore, in some injury models, its efficacy is even better than that of carbenoxolone and cimetidine (Rajabian, et al., 2022). Finally, the efflux of K^+^ ions after opening blocks the voltage-sensitive Ca^2+^ channels, which ultimately results in the dilation of blood vessels and improvement of smooth muscle contraction, thereby increasing GMBF.

Additionally, TRPV1 is a novel type of ion channel that has been demonstrated to mollify GMI, so it has the potential to become a promising target for various TCMs (Tashima, et al., 2020). A case in point is curcumin, an agonist that activates the TRPV1 receptor in a dose-dependent manner. Afterwards, the activated TRPV1 receptor increases the excitability of the endogenous release of calcitonin gene-related peptide (CGRP) from sensory nerves, expediting the release of PGs and NO from the CGRP to dilate blood vessels, ultimately reduce the ischemic area (Czekaj, et al., 2018; Ma, et al., 2021). Furthermore, Koji discovered that the PGE2 produced by COX-1 interacts with ASIC3-mediated sensory neurons through EP1 receptors to increase GMBF, which provides a novel treatment strategy for GI diseases (Takeuchi, et al., 2015). In addition to the above benefits, five Chinese drugs from *Alpinia* Roxb. (which contain *Alpinia officinarum* Hance, *Alpinia galanga* (Linn.), *Alpinia katsumadai* Hayata, *Alpinia galangal*, *Alpinia oxyphylla*) remarkably strengthen GMBF in all parts of the gastric antrum, including the anterior and posterior wall as well as great and minor curvature, to regulate “viscosity”, “thickening” and “coagulability” of the blood (Qin, et al., 2020), thereby unleashing positive effects for GMBF to effectively fulfill its role as a transportation hub. Overall, we exemplify the manners and mechanisms of GMBF recovery mediated by TCMs and discuss targets of ECs as well as Endothelium-derived factors produced by ECs such as NO and PGE_2_ during the above processes.

## 5 Repairs of the lamina propria barrier

**FIGURE 3:**
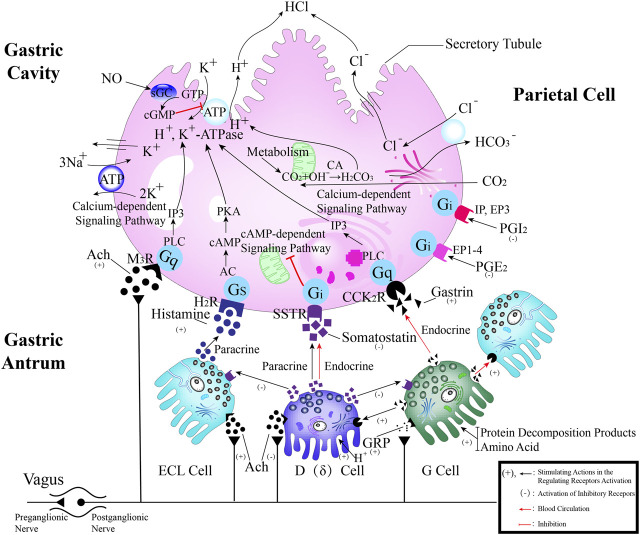
The process of secreting gastric hydrochloric acid secretion by parietal cells involves the active transport of H^+^ to the cellular endocrine tubules. Additionally, H^+^/K^+^ pumps, also known as proton pumps, within the secretory tubules and vesicles work to pump H^+^ from the parietal cells into the fundus gland cavity against the concentration gradient. The results in the formation hydrochloric acid, which can be stimulated by either neural or humoral factors through the respective receptors on the parietal cells, or indirectly through interactions with on other. Abbreviations: ECL cells: Enterochromaffin-like cells; D (δ) cells: Named after their morphology, which resembles pancreatic islet D cells; G cells: Secreting gastrin/gastrin (GAS); CA: Carbonic anhydrase; Ach: Acetylcholine; histamine: HA; GRP: Gastrin releasing peptide; M3 receptor: Muscarinic receptor 3; H_2_ receptor: Histamine receptor; SST receptor: Somatostatin receptor; CCK_2_ receptor: Gastrin/Cholecystokinin receptor.

Eighty percent of the lamina propria is composed of acid-secreting glands, including parietal cells and chief cells. Hydrochloric acid secreted by parietal cells generates the highly acidic environment of the gastric lumen (pH < 2), which effectively kills bacteria, facilitates food digestion, activates pepsinogen (PG) to pepsin and enhances the absorption of minerals such as phosphate, calcium, and iron (Engevik, et al., 2020). Correspondingly, GMI is a multifactorial and complex disease involving disruption and imbalance of the above physiological functions, so the occurrence of counterdiffusion of acid damage refers to the major mechanism underlying GMI and ulcer formation. Many researchers have presented evidence to prove that TCMs moderate hydrochloric acid secretion and the lamina propria barrier by regulating GPCR-mediated receptor pathways (e.g., Ach, HA, GAS, CCK, SST, PGE_2_, PGI_2_) (Câmara Neto, et al., 2022), ion channels or transporters (including H^+^/K^+^-ATPase, K^+^ channel, Na^+^/K^+^-ATPase, Ca^2+^-ATPase (SERCA), Cl^−^ channel, Cl^−^/HCO_3_^−^ exchanger, H^+^/Na^+^ exchanger, etc.), NO, vagus nerve, parietal cell-mediated PG, and other antagonists or enhancers (Figure 3), thereby regulating the physiological activity of parietal cell acid secretion.

### 5.1 Receptor pathway blockers of TCMs for overproduction of hydrochloric acid

In terms of receptor pathways, the key intermediate is cAMP, which serves as a second receptor. TCMs can modulate cAMP, thereby regulating the physiological synthesis of hydrochloric acid initiated by neurotransmitters and humoral factors as well as PG, which are two key factors involved in hyperacidity induced by GMI. A study noted that a mixture of patchouli and tangerine peel essential oils (EO), with a ratio of 1:2, significantly reduced HA expression is known to inhibit cAMP activity, which helps alleviate the expansion of the endoplasmic reticulum induced by parietal cell activation, reduces of secretory tubules activity, slows the release of PGC caused by the destruction of mitochondrial morphology of chief cells, and ultimately improves GMI to safeguard GM (Chen, et al., 2022). Improved regulation and balance of gastric acid secretion has been identified by mutual crosstalk alleviation between receptor pathways. Further studies have substantiated that EO of patchouli and tangerine peel noticeably decreased the expression of GAS and the proportion of cAMP/cGMP via the JNK signaling pathway. And low expression of endogenous action molecules that mediate HA is found in it, thus, the EO has exhibited exceptional efficacy in repairing mucosal damage (Chen, et al., 2022). It follows that facilitating the coordination among diverse endogenous molecules in the receptor pathway is crucial to regulate the secretion of hydrochloric acid in parietal cells.

### 5.2 PPIs of TCMs for overproduction of hydrochloric acid

#### 5.2.1 H^+^/K^+^-ATPase

Compared to the receptor pathways of acid secretion, studies have determined that TCMs are more effective in enabling antisecretory therapy. This therapy involves targeting the ion channels or transporters in parietal cells, which is the main strategy for treating GMI and preventing its complications (Sverdén, et al., 2019; Deng, et al., 2021; Maev et al., 2022). H^+^/K^+^-ATPase is the dominant rate-limiting factor in the final step of parietal cells secreting hydrochloric acid (Matsukawa, et al., 2018). Proton pump route has proven to be of the most preferred and common approach for the restoration of GMI by TCMs. It is worth noting that *Hypericum perforatum* exerts remarkable activity against acid hypersecretion, and it has a strong affinity for the inhibitor binding site and H^+^, K^+^ recognition site of the catalytic subunit α in H^+^/K^+^-ATPase, as reported in previous studies (Shin, et al., 2011; Zhang, et al., 2020b; Sofi, et al., 2020). And this study demonstrated that *Hypericum perforatum* is nearly as effective as esomeprazole in preventing ethanol induced GU and has greater binding affinity to PPIs than esomeprazole (Sofi, et al., 2020). Molecular docking experiments further revealed that taxifolin can regulate enzyme activity by binding with Tyr801, a classic conserved site in H^+^/K^+^-ATPase, and its polar residues reside in the dynamic network of hydrogen bonds. With the administration of 10 mg/kg of taxifolin, the ulcer area was reduced by 40.5%, equivalent to the ulcer healing effect of 20 mg/kg of omeprazole (Moura, et al., 2021).

#### 5.2.2 K ion channels

Moreover, several types of K ion channels, serving as alternative targets of H^+^/K^+^-ATPase can precisely and rapidly inhibit of acid secretion independently of pump activity, which can be achieved by regulating Ca^2+^-ATPase or by competitively inhibiting H^+^/K^+^-ATPase activity. For example, a study has verified that Na^+^/K^+^-ATPase ion channel corresponds with H^+^/K^+^-ATPase in NEO and lessens parietal cell secretion by competitively combining with K^+^ to restrain K^+^ exchange in H^+^/K^+^-ATPase (Mazzoni, et al., 2021), as supported by the considerable homology (∼60% identity) of the H^+^/K^+^-ATPase α subunit with the Na^+^/K^+^-ATPase catalytic α subunit. Hence, many researchers believe that Na^+^/K^+^-ATPase can act as a valid marker for oxyntopeptic cells, and it is expected to become a new and prospective alternative target for drugs to treat GMI. In addition, the α subunit in H^+^/K^+^-ATPase exhibits a fairly high degree of homology among animal species, up to 90% (Shin, et al., 2011). Another study has confirmed that burdock (*Arctium lappa* L.) blocks the GAS-stimulated acid secretion by inhibiting K^+^ efflux induced by the K^+^ channel, which is related to blocking Ca^2+^ inflow into parietal cells or decreasing Ca^2+^ release from the endoplasmic reticulum, thereby keeping H^+^/K^+^-ATPase in an inactive and dephosphorylated state. The inhibitory activity of burdock is stronger than ranitidine at the same concentration and is comparable to omeprazole (Silva, et al., 2018). In addition, a study evidenced that as the rate of H^+^/Na^+^ exchange decreased, the average pH of gastric juice subsequently decreased as well (Aggarwal, et al., 2021). From the above studies on parietal cells, it can be inferred that TCMs ameliorate alterations and dysregulation in gastric acid secretion through the modulation of agonists, antagonists and ion channels that stimulate parietal cell activity.

## 6 Toxicological effect of TCMs on the gastric mucosal barrier

Although TCMs and their derivatives have long been believed to be safe and have low toxicity for the treatment of GMI, recent discoveries suggest that some TCMs may aggravate GMI symptoms through their forms of existence, compatibility, metabolic absorption path, concentration, and route of administration. Some TCMs may even cause harm to the human body, such as liver injury. The composition of TCMs is intricate and contains multiphase systems during the process of decocting and boiling, such as true solutions, colloidal solutions, emulsions, suspensions, and more. The components of drugs may exist in various forms within a heterogeneous system, and they can affect GMI in diverse ways, including adverse effects for disease treatment. These forms of medicine may include alkaloids, glycosides, phytotoxic proteins, terpenes and lactones, anthraquinones, heavy metals, among others (Pan, et al., 2020). For example, salicylic acid, a common over-the-counter gel used for relieving oral ulcers (which is a source of salicylate), can lead to the development of refractory GI ulcers. However, when combined with a variety of drug components, salicylic acid can be developed into an anti-gastric ulcer medication. A study found that horseradish powder and extract are rich in natural antioxidants, such as polyphenols and flavonoids (salicylic acid, quercetin, ellagic acid, chlorogenic acid catechol and coffee), which provide an anti-ulcer effect on indomethacin-induced peptic ulcer, and are expected to be useful in the treatment of GU (Wahba and Shelbaya, 2018).

Along with its distinct form of existence, drug components have the potential to induce toxicity. They can be converted into toxic substances, regulate the expression of receptors and metabolic enzymes, and affect the metabolic and absorption pathways of the human body, forming a drug-target-dynamic/toxic response network (Wei, et al., 2021) that may harm to the human body while treating diseases. For example, once absorbed by the GI tract, gambog can regulate the mRNA and protein expression of AQP3 and AQP4, resulting in a significant increase. This, in turn, leads to hyperemia and edema of the GM, ultimately causing GMI (Zhao, et al., 2016). At present, researchers have reduced the toxicity of drugs on the human body by improving the extraction and purification process of TCMs, prolonging the rate of drug absorption, selecting appropriate drug doses, adjusting compatibility ratio, and employing chemical modification. For example, when 0.30% (m/v) xanthan gum and 0.20% (m/v) siraitia grosvenorii saponin are added to the oral liquid of the herbal prescription (consisting of *Dendrobium candidum*, Chinese wolfberry and puerarin), the resulting oral liquid displays excellent stability along with the highest overall acceptable sensory score. Furthermore, studies have confirmed that the oral liquid is effective in significantly preventing the formation of the GU within a few days (Pei, et al., 2021). We do not want the situation where the disadvantages of the above-mentioned drugs outweigh the benefits. Therefore, when developing and designing drugs, it is important for researchers to consider various factors. These include the existing form, compatibility, metabolic absorption pathway, concentration, and administration route of drug components. Additionally, it is crucial to timely discover and avoid any potential toxicity of the drugs.

## 7 Conclusion

**FIGURE 4:**
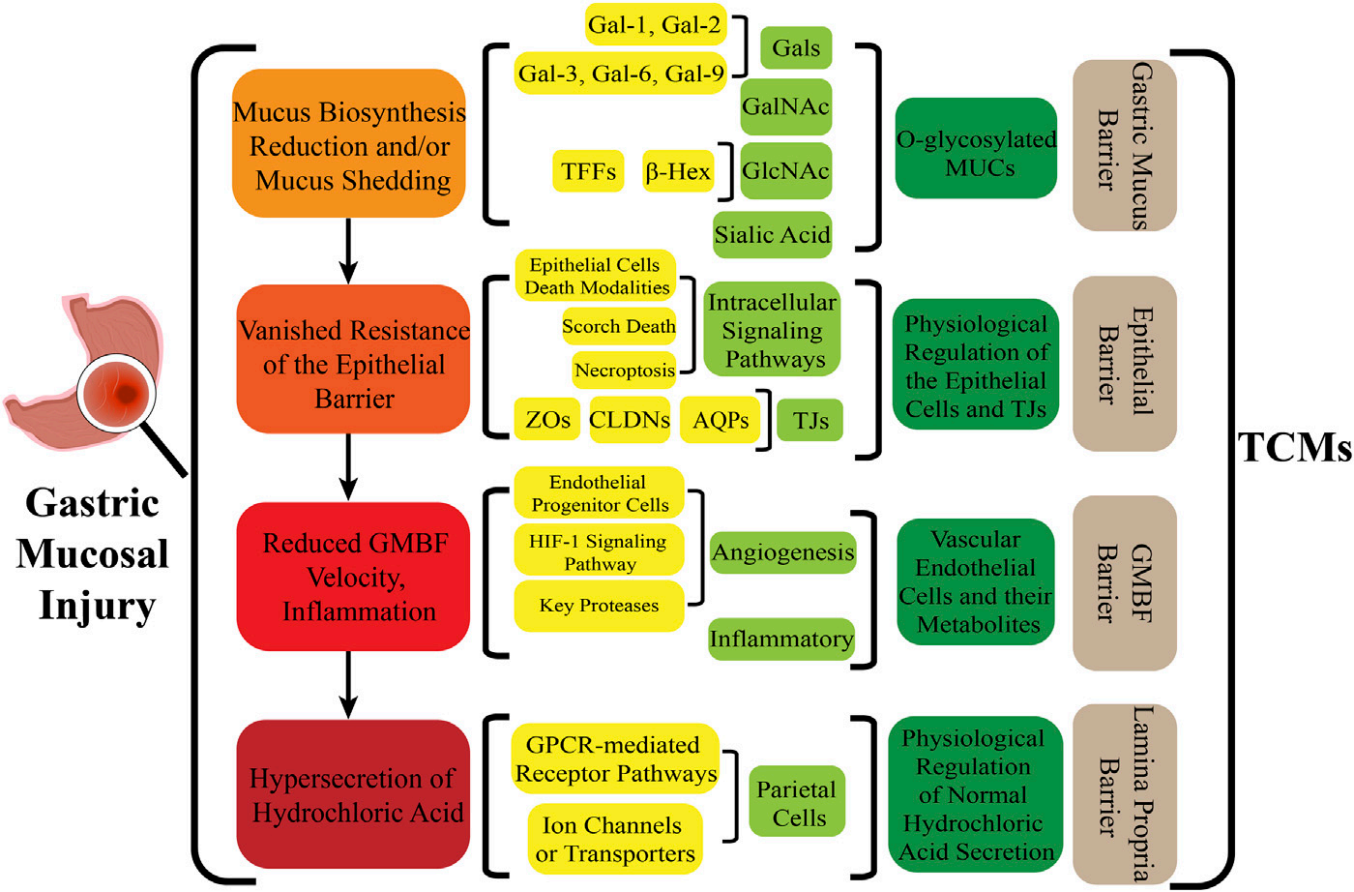
Pharmacological mechanism of TCMs in repairing the gastric mucosal barrier.

Analyzing the manners in which complementary and alternative TCMs act on the main components of the GM and targeting key components with molecular methods have revealed targeted pharmacological effects on GMI in this review (Figure 4). This leads to a practical and feasible approach to assess the curative effects of TCMs for treating disorders of the GMI. Abundant pharmacological evidence has proven that TCMs and their derivatives be of natural/replacement therapy for various diseases including GMI, which has been widely applied in the clinical treatment of patients and has achieved significant advancements in superior efficacy. These TCMs, insects and their derivatives for instance, containing Kangfuxin (an ethanol extract of *Periplaneta americana* L. (Dictyoptera; Blattidae)), insect tea (such as *Hydrillodes morosa* (Butler) and *Aglossa dimidiate* (Haworth)) and natural honey. As for single Chinese medicine and compound of TCMs, which consists of *Astragalus membranaceus* (Fisch.) Bunge, Chaihu Shugan powder (CSP), Huangqi Jianzhong Tang (HQJZT) and others. Besides that, fungi-based products in TCMs include polysaccharides purified from *Hericium erinaceus*, *Auricularia polytricha* and *Flammulina velutipes*, among others. Moreover, some TCMs belong to the category of “medicinal food” that has officially entered the stage of legal management since November 2021, which is less harmful to the human body and can rely on food to prevent and treat diseases. In these analyses, it is not hard to appreciate that the multiple targets, multiple pathways, low toxicity, and multiple levels, along with great drug development potential of TCMs characteristics can exert reparative effects on GMI either independently or in combination with other TCMs.

The research focuses of this article on how TCMs target the gastric mucosa barrier to improve GMI. However, thus far, various chemical constituents or forms of TCMs that aid in the repair of mucosal injury have not been adequately described in this review. Moreover, specific types of TCMs corresponding to specific mucosal repair effects are also the focus of research, which are closely linked to determining the repair effects of certain TCMs and exploring the development of targeted drugs. In addition, this paper evaluates the efficacy of TCMs and their derivatives in repairing mucosal damage, but there is still limited experimental data and incomplete literature pose certain challenges. Furthermore, there is a lack of sufficient data on the toxicity, pharmacokinetics, and clinical studies of TCMs. Although TCMs can effectively benefit the treatment of GMI, the potential side effects associated with long-term use should be considered, just like with western medicines. Finally, the industrial development and clinical application of TCMs are limited due to its insufficient in-depth research, incomplete evaluation, vague explanation and un unclear mechanism of action. Based upon these facts, there is an urgent requirement to identify alternative targets or key enzymes, facilitate the mitigation of drug toxicity, dose and side effects, as well as determine and evaluate the effectiveness for the development and utilization of TCMs by individuals.
